# Rapid development of a decision-aid for people with dementia and their families during COVID-19

**DOI:** 10.1192/j.eurpsy.2021.277

**Published:** 2021-08-13

**Authors:** E. West, N. Kupeli, K. Moore, E. Sampson, N. Aker, P. Nair, N. Davies

**Affiliations:** 1 Division Of Psychiatry, University College London, Marie Curie Palliative Care Research Society, London, United Kingdom; 2 Research Department Of Primary Care And Population Health, Centre for ageing population Studies, London, United Kingdom

**Keywords:** COVID-19, Decision-making, Place of Care / Place of Death, Advance Care Planning

## Abstract

**Introduction:**

COVID-19 as a pandemic has disproportionately affected older adults, including those with dementia. The effects on health and social care systems has necessitated a rapid-response approach to care planning and decision-making in this population, with reflexivity and responsiveness to changing individual and system needs at its core. In light of this, a decision-making tool to help families of persons with dementia was developed using a combination of qualitative data and evidence synthesis.

**Objectives:**

To develop a decision-aid using a combination of assessment and evidence-gathering methods for families of persons with dementia.

**Methods:**

Semi-structured interviews with helpline staff from national end-of-life and supportive care organisations formed the basis of the tool design. Co-design with people living with dementia, current and former carers and experts in general practice and social care shaped the next stage. Simultaneously, a rapid review of current evidence on making decisions with older people at the end of life was undertaken.

**Results:**

Output from interviews covered many topics, including trust, agency and confusion in making decisions in the context of COVID-19. The rapid review of existing evidence highlighted the need to consider both process and outcome elements of decision-making.

**Conclusions:**

Combining different sources and forms of evidence was efficient and valuable in creating a novel decision-making tool for persons with dementia and their families within the context of COVID-19. The decision-aid covered care planning, caregiver support systems, access to information and contingency considerations. Upon publication, the tool was adopted by NHS England and other leading healthcare organisations.

**Disclosure:**

No significant relationships.

